# Supplementary material for: Bimodal distributions of anti-Trypanosoma cruzi antibody levels in blood donors are associated with parasite detection and antibody waning in peripheral blood
Source: PLoS Negl Trop Dis. 2025 May 30;19(5):e0012724. doi: 10.1371/journal.pntd.0012724 (PMC12124490; doi:10.1371/journal.pntd.0012724)

**Supporting information**

**S1 Fig. Correlation of antibody levels at visit 1 with *T. cruzi* PCR results**

**Figure 1 supplemental**

Legend S1: Scatter plots of signal-to-cutoff (S/C) values at visit 1

CMIA/Abbott: Chagas Architect, Abbott, Germany; EIA Lysate: ELISA Lisado, Wiener Lab., Argentina; Recombinant EIA: ELISA Recombinante, Wiener Lab., Argentina; Vitros: Vitros Immunodiagnostics Products Anti-T. cruzi (Chagas) Assay (Ortho Clinical Diagnosis, Raritan NJ, USA.


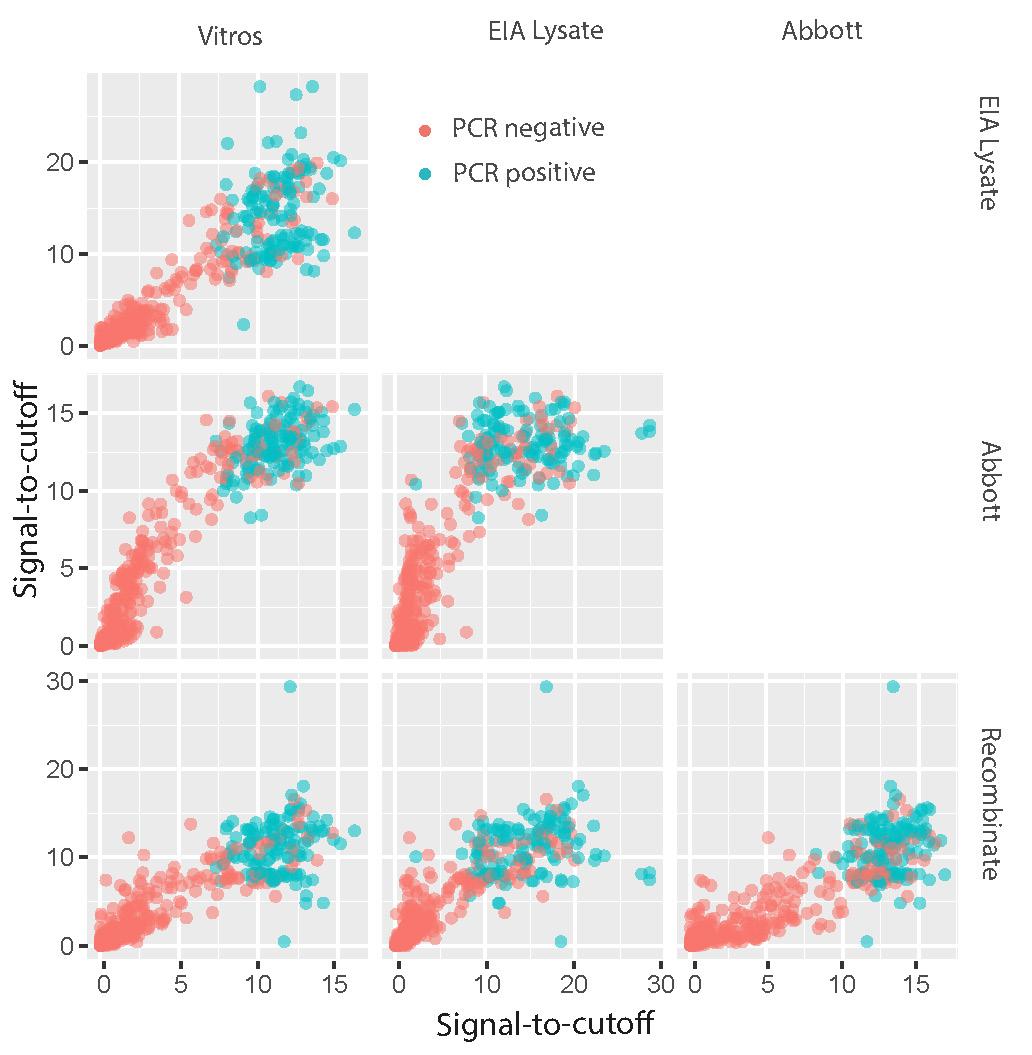

Supplement: S1 Fig — Fig 1 supplemental. Legend S1 Fig: Scatter plots of signal-to-cutoff (S/C) values at visit 1. CMIA/Abbott: Chagas Architect, Abbott, Germany; EIA Lysate: ELISA Lisado, Wiener Lab., Argentina; Recombinant EIA: ELISA Recombinante, Wiener Lab., Argentina; Vitros: Vitros Immunodiagnostics Products Anti-T. cruzi (Chagas) Assay (Ortho Clinical Diagnosis, Raritan NJ, USA. (DOCX) [file pntd.0012724.s001.docx]
